# Design and Optimization of High-Amplification-Ratio Micromanipulator Based on Compliant Mechanism

**DOI:** 10.3390/mi17080883

**Published:** 2026-07-24

**Authors:** Feng Zhao, Wentao Huang, Jianguo Liao, Xiaodong Chen, Fengchen Zhai, Xueyan Chen

**Affiliations:** 1 School of Mechanical Engineering, Shenyang Ligong University, Shenyang 110159, China; 2 Institute of International Engineering, Shenyang Ligong University, Shenyang 110159, China; 3 School of Equipment Engineering, Shenyang Ligong University, Shenyang 110159, China; 4 School of Aerospace, Harbin Institute of Technology, Harbin 150080, China

**Keywords:** micromanipulator, compliant mechanism, three-stage DA, piezoelectric actuator, structure size optimization

## Abstract

Displacement magnification (DM) is one of the key indicators to measure the performance of a micromanipulator. Based on the principle of triangle amplification, a series of three-stage displacement amplification (DA) micromanipulators is designed in this paper, which is driven by a piezoelectric actuator. The mechanism is composed of a compound rhombus mechanism, a bridge mechanism and two parallelogram mechanisms. The compound rhombus mechanism is located at the front end and has high stiffness characteristics. The bridge mechanism is located at the back end and is connected to the parallelogram mechanism to realize the parallel output of the clamping end. The DM model of the mechanism is established. On this basis, the structural size optimization design is carried out. With the goal of maximizing the DM, the key geometric parameters such as the thickness and length of the hinge and the section size of the key rod are selected as the design variables. Considering the constraints of material strength and input stiffness, the parameters of the micromanipulator are optimized. The correctness of the optimization method is verified by finite element simulation and experimental test. The results show that the DM of the optimized micromanipulator is significantly improved under the premise of maintaining sufficient stiffness, which can effectively guide the performance improvement and structural design of the micromanipulator.

## 1. Introduction

At present, micro-operation systems are widely used in high-precision fields such as minimally invasive surgery, optical fiber precision alignment, and scanning probe microscopic detection [1,2,3,4]. As the end-effector of a micro-operation system, a micromanipulator is typically required to perform operations such as gripping and transporting [5]. Advances in intelligent sensing and autonomous state perception have been further integrated into compliant mechanisms. The fine-tuned multimodal large language model proposed by Mao et al. [6] is applied to the shape recognition system of tensegrity structures equipped with flexible sensors, which enables real-time state estimation and adaptive closed-loop control. In the field of optical fiber alignment, the diameter of micro and single-mode optical fibers can be as small as 1 μm, while the diameters of multimode and large-diameter optical fibers span several hundred micrometers, and the alignment accuracy of the fiber core is required to be at the submicron or even nanometer level. Therefore, the micromanipulator should possess advantages such as a large gripping range, high precision, fast response, translational gripping, and compact structure to handle objects with irregular shapes, large dimensions, and susceptibility to damage, thereby meeting the demands of complex micro-operation tasks [7,8].

Piezoelectric ceramic actuators exhibit advantages such as high positioning resolution, high thrust, no electromagnetic interference, and low energy consumption, making them a preferred choice for precision positioning; however, their stroke is short, with the total stack elongation being approximately 0.1–0.15% of the actuator length [9,10,11]. To overcome this inherent drawback, micromanipulators integrate compliant DA mechanisms to amplify the output displacement of the piezoelectric actuator. Compliant mechanisms transmit motion and force through the elastic deformation of flexure hinges, offering benefits such as frictionlessness, backlash elimination, and assembly-free construction [12,13,14]. Common amplification mechanism configurations include bridge-type, rhombic-type, lever-type, and Scott-Russell mechanisms, among which the bridge-type and rhombic-type mechanisms are widely adopted due to their structural symmetry and high stiffness. By connecting basic amplification units in series or parallel, multistage amplification mechanisms can be constructed to further enhance the displacement amplification ratio (DAR) [15,16,17,18]. Chen et al. [19] proposed a dual-constrained single-stage DA mechanism design that effectively improves the output accuracy of the mechanism, achieving an output DAR of 5.2. Das et al. [20] presented a symmetrically designed piezoelectric-actuated compliant microgripper featuring a two-stage displacement transmission and amplification mechanism: two lever-type mechanisms symmetrically connected to a bridge-type mechanism jointly amplify the output displacement of the piezoelectric actuator, resulting in a DAR of 11.19. Das et al. [21] proposed a compact piezoelectric microgripper integrating a three-stage amplification and transmission mechanism, in which a bridge-type mechanism is connected in series with two lever-type mechanisms to form a three-stage amplification chain, with parallelogram mechanisms incorporated to achieve translational gripping. The DAR is 20.1, and the output displacement is 140 μm. In another study, Das et al. [22] designed a high-precision piezoelectric microgripper based on a three-stage DA mechanism, where a bridge-type mechanism with two output ports is connected in series with two successive lever mechanisms, and the output motion is linearized by a parallelogram mechanism. Through finite element analysis and experimental validation, the total DAR is 12.76.

A review of the above research progress reveals a significant stepwise increase in DAR, from single-stage amplification (5.2) to two-stage amplification (11.19) and further to three-stage amplification (20.1). However, the enhancement of the amplification ratio is not merely a matter of increasing the number of stages but relies on rational configuration optimization design, stiffness matching strategies among the stages, and optimization of key dimensional parameters. This evolutionary pattern provides an important reference for the high-performance design of subsequent microgrippers. Zhao et al. [23] designed a three-stage amplified piezoelectric-driven microgripper with displacement and gripping force sensing capabilities. In that study, key structural parameters were determined empirically, and finite element analysis was employed to optimize and adjust the parameters to maximize output displacement while ensuring that stresses remained within safe limits. Theoretical modeling was used to derive the amplification ratio, and finite element simulation was conducted to validate the design effectiveness. The DAR of this micromanipulator is 33.6. Wang et al. [24] reported a three-stage compliant amplification mechanism in which, from the optimization strategy perspective, key geometric parameters affecting amplification ratio, stress distribution, and natural frequency were identified through analytical modeling, and design parameters were iteratively optimized using finite element analysis. Experimental results demonstrate that the optimized micromanipulator achieves a maximum output stroke of 190 μm and a DAR of 22.8 under a driving voltage of 100 V, with a working modal frequency of 953 Hz, validating the effectiveness of the optimization design. In the optimization design presented in this paper, to reveal the influence of structural parameters on the performance of the micromanipulator, the response surface methodology is adopted to establish a mathematical model between the parameters and the responses. Finite element analysis is employed to evaluate the performance responses within the design space, and multi-objective optimization is performed based on the response surface model.

In summary, this paper presents the structural design and optimization design of a high-amplification-ratio three-stage amplified micromanipulator. The mechanism is driven by a piezoelectric ceramic actuator and consists of a compound rhombus mechanism, a bridge-type mechanism, and two parallelogram mechanisms connected in series to form a three-stage DA chain, achieving efficient DA while maintaining translational gripping. In terms of optimization design, unlike the empirical trial-and-error or single-parameter finite-element iterative optimization methods employed in existing studies, this paper adopts the response surface methodology to establish high-precision surrogate models relating key geometric parameters to the mechanism performance responses. Based on these models, multi-objective optimization is performed with the maximization of the DAR as the primary optimization objective, subject to material strength constraints, to identify the optimal design configuration.

## 2. Mechanical Design and Analysis

### 2.1. Series Design of Triangle Amplification Principle

**Figure 1:**
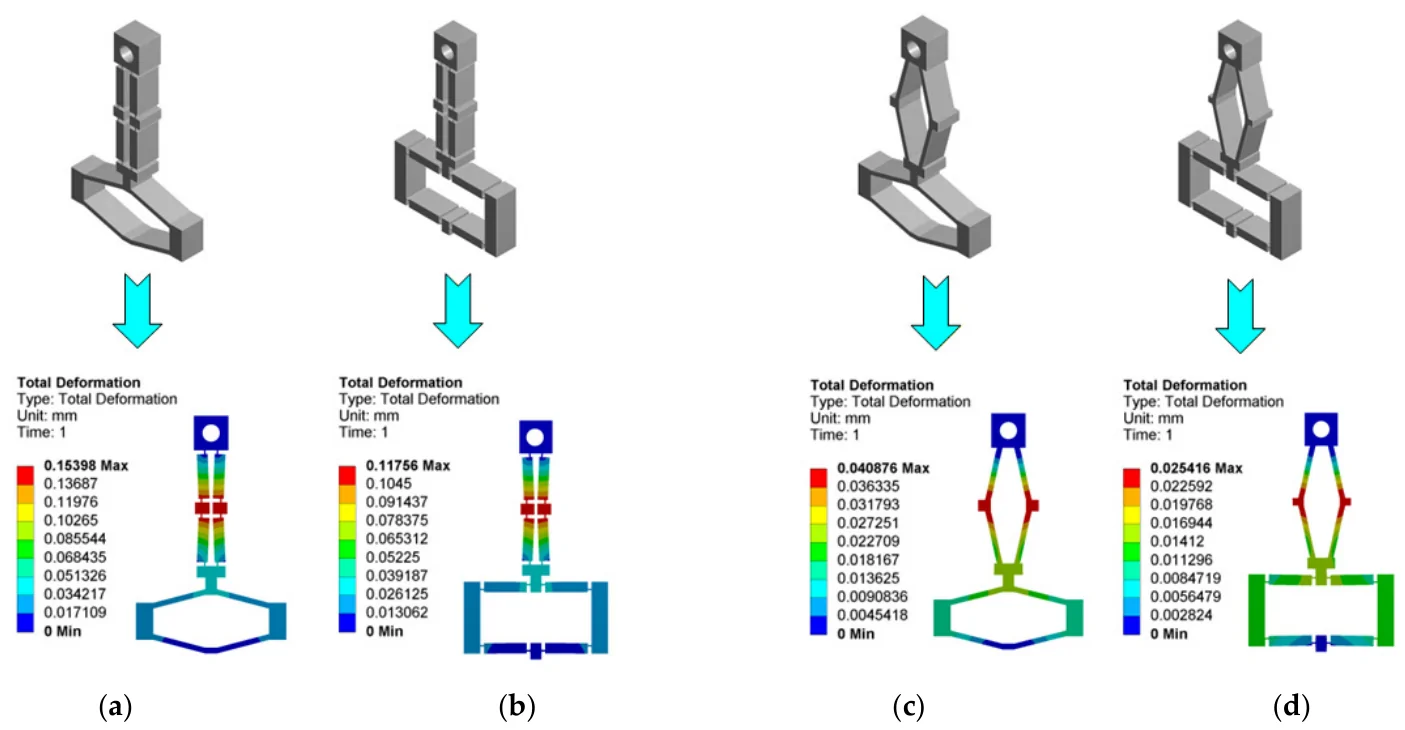
Series design of triangle amplification principle: (**a**) rhombus-bridge serial amplification configuration, (**b**) bridge-rhombus serial amplification configuration, (**c**) dual-rhombus serial amplification configuration, and (**d**) dual-bridge serial amplification configuration.

Both rhombus-type and bridge-type amplification mechanisms are typical structural implementations of the triangular displacement amplification principle. This work investigates the coupling characteristics of cascaded amplification units derived from this principle. Four configurations of two-stage flexure amplification mechanisms are presented in Figure 1, including rhombus-bridge cascade, dual-rhombus cascade and dual-bridge cascade layouts. The displacement amplification ratio (DAR) of each configuration is acquired via finite element deformation analysis: the DAR values of Figure 1a, Figure 1b, Figure 1c and Figure 1d are 15.398, 11.756, 4.08 and 2.54, respectively. Simulation results validate that the cascade configuration with a rhombus amplifier as the first stage and a bridge amplifier as the second stage achieves the optimum displacement amplification performance. This further demonstrates that stiffness impedance matching between preceding and subsequent amplification units is critical to the overall amplification capacity of cascaded mechanisms designed with the triangular amplification principle.

### 2.2. Mechanism Magnification Analysis

**Figure 2:**
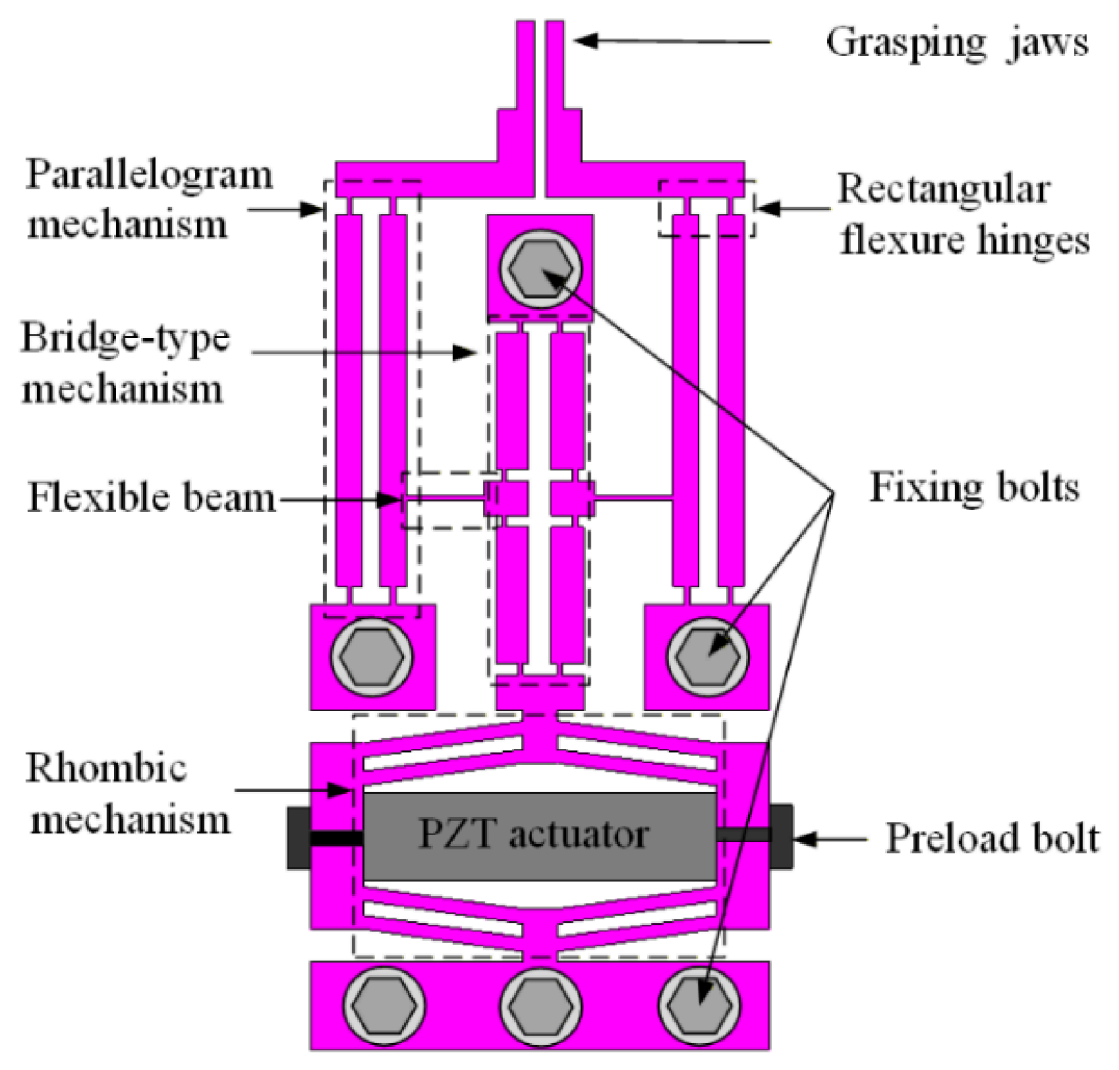
Structural diagram for micromanipulator.

The overall structure of the three-stage DA micromanipulator is shown in Figure 2. A piezoelectric ceramic actuator is employed as the driving unit, and a preload bolt is used to apply an appropriate preload force to the actuator to ensure its normal operation. Fixing bolts are adopted to reliably mount the entire mechanism onto a base. The DA chain is constructed by connecting three stages in series: the first stage is a rhombus mechanism, which possesses high stiffness characteristics and provides preliminary amplification of the initial displacement output by the piezoelectric actuator; the second stage is a bridge-type mechanism, which receives the displacement transmitted from the rhombus mechanism and further amplifies it; the third stage is composed of a parallelogram mechanism and gripping jaws, which not only realizes the final-stage DA but also ensures that the jaws perform a translational motion, thereby avoiding shear damage to the manipulated object. The stages are connected and motion is transmitted through flexible beams; rectangular flexure hinges serve as key elastic elements, providing repeatable, precise deformation and guiding the motion direction. This three-stage series configuration achieves multistage efficient DA while maintaining a compact structure and translational output at the gripping tip.

## 3. Mechanical Design and Analysis of the Micromanipulator

### 3.1. DAR Modeling

**Figure 3:**
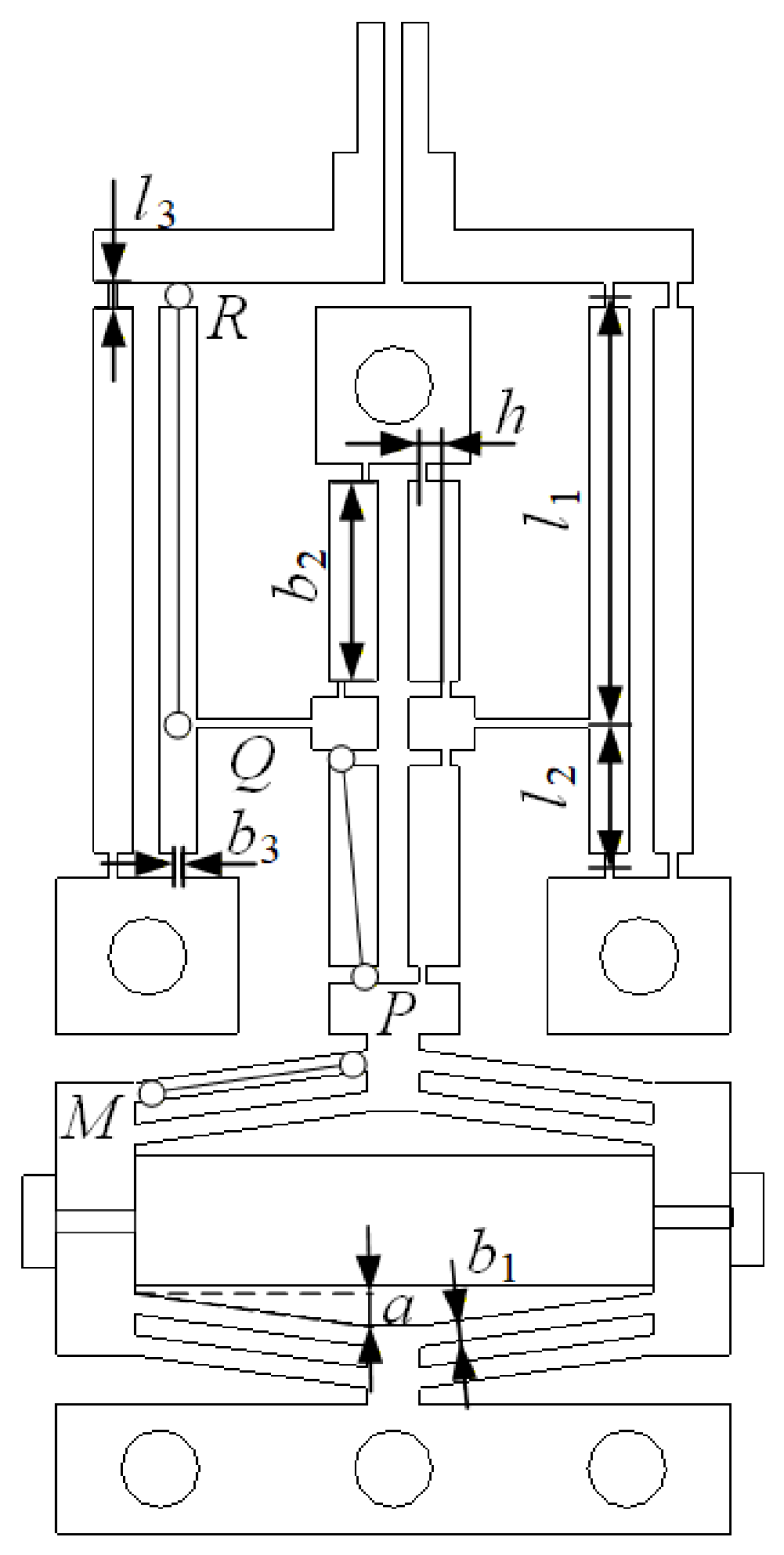
Schematic diagram of the micromanipulator.

Only small elastic deformation occurs during the working process of the flexure hinge, and all the flexibility matrices are established based on the linear elastic small deformation theory. The motions of all mechanisms are limited in the *xy* plane (rotating around the *z*-axis), and the out-of-plane deformation coupling is not considered. As shown in Figure 3, for a rectangular flexure hinge, the compliance matrix in its local coordinate system is given by:

(1) $$C_{hing}=\left[\begin{array}{ccc} C_{11} & 0 & 0\\ 0 & C_{22} & C_{23}\\ 0 & C_{32} & C_{33} \end{array}\right]=\left[\begin{array}{ccc} \frac{l}{Ebt} & 0 & 0\\ 0 & \frac{4l^{3}}{Ebt^{3}} & \frac{6l^{2}}{Ebt^{3}}\\ 0 & \frac{6l^{2}}{Ebt^{3}} & \frac{12l}{Ebt^{3}} \end{array}\right]$$

where *l* denotes the hinge length, *b* denotes the hinge width, *t* denotes the minimum hinge thickness, and *E* denotes the elastic modulus.

When it is necessary to transform force and displacement from one coordinate system to another:

(2) $${\left[AD\right]}_{ij}\;=\left[\begin{array}{cc} R_{ij} & 0\\ D_{ij}R_{ij} & R_{ij} \end{array}\right]$$

Let the rotation angle of coordinate *j* relative to coordinate *i* be *θ* and the relative translation of origin be (*dx*, *dy*).

The adjoint transformation matrix *Ad_ij_* (3 × 3 matrix) is used to transform the force and displacement from one coordinate system to another coordinate system. For plane problems (rotating around the z-axis and moving in the x y plane), the transformation matrix is:

(3) $$R_{ij}=\left[\begin{array}{ccc} \cos\theta & -\sin\theta & 0\\ \sin\theta & \cos\theta & 0\\ 0 & 0 & 1 \end{array}\right],\;D_{ij}\;=\left[\begin{array}{ccc} 0 & -dz & dy\;\\ dz & 0 & -dx\\ \;\;-dz & 0 & dx \end{array}\right]\;\;$$

When transformed to a new coordinate system, the compliance matrix is transformed as:

(4) $$C_{j}\;={\left[Ad\right]}_{ij}^{T}\;C_{i}\;{\left[Ad\right]}_{ij}\;$$

The diamond mechanism has a central symmetrical structure. In order to simplify the analysis, a quarter model is used for research. As shown in Figure 3, point *M* is the input force point, and point *P* is the output point. From the input point *M* to the fixed end, the kinematic chain consists of two series of flexible hinges. In the *M*-point coordinate system, the equivalent flexibility matrix of the quarter-model is:

(5) $$C_{1/4}\;={\left[Ad\right]}_{M1}^{T}\;C_{hinge1}\;{\left[Ad\right]}_{M1}\;+{\left[Ad\right]}_{M2}^{T}\;C_{hinge2}\;{\left[Ad\right]}_{M2}\;$$

Using symmetry, the overall compliance matrix of the rhombus mechanism is given by:

(6) $$C_{rho}\;=2\cdot T_{sym}\;C_{1/4}\;T_{sym}^{T}\;\;\;\;\;$$

When a force *F*_in_ (applied along the x-direction) is exerted at the input point *M*, the displacement at the output point *P* is:

(7) $$\left[\begin{array}{c} u_{in}\;\\ u_{mid}\\ \theta_{out} \end{array}\right]\;=C_{dia}\left[\begin{array}{c} F_{in}\\ 0\\ 0 \end{array}\right]\;\;\;\;\;$$

Thus, the amplification ratio of the rhombus mechanism is:

(8) $$A_{rho}\;=\frac{\;u_{mid}}{u_{in}}\;=\frac{\;{\left[C_{dia}\right]}_{21}}{{\left[C_{dia}\right]}_{11}}\;\;$$

The bridge-type mechanism has two ports: input port *P* and output port *Q*. Its force–displacement relationship is expressed as:

(9) $$\left[\begin{array}{c} \epsilon_{in2}\\ \epsilon_{out2} \end{array}\right]=\left[\begin{array}{cc} C_{bridge}^{11} & C_{bridge}^{12}\\ C_{bridge}^{21} & C_{bridge}^{22} \end{array}\right]\left[\begin{array}{c} F_{in2}\;\\ F_{out2} \end{array}\right]$$

where *ϵ*_in2_ denotes the displacement at input port *P* (*u_in_*_2_, *v_in_*_2_, *θ*_*_in_*_2_), *ϵ_out_*_2_ denotes the displacement at output port *Q* (*u_out_*_2_, *v_out_*_2_, *θ_out_*_2_), *F_in_*_2_ denotes the external force applied at input port *P*, and *F_out_*_2_ denotes the external force applied at output port *Q*.

Then, the amplification ratio of the bridge-type mechanism is:

(10) $$A_{bri}\;=\frac{\;u_{out2}}{u_{in2}}\;=\frac{\;{\left[C_{bri}^{21}\right]}_{11}}{{\left[C_{bri}^{11}\right]}_{11}}\;\;$$

The parallelogram mechanism consists of two identical branches connected in parallel. For a single branch (from input port *Q* to output port *R*):

(11) $$C_{leg\;}={\left[Ad\right]}_{Q1}^{T}\;C_{h1}\;{\left[Ad\right]}_{Q1}\;+{\left[Ad\right]}_{Q2}^{T}\;C_{h2}\;{\left[Ad\right]}_{Q2}\;$$

The two branches are completely identical, and after parallel connection:

(12) $$C_{para}\;=\frac{1}{2}\;C_{leg}\;$$

The force–displacement relationship of the parallelogram mechanism is:

(13) $$\epsilon_{final}\;=C_{para}\;F_{out3}\;$$

The displacement at the output port P of the first stage (rhombus) serves as the input displacement of the second stage (bridge-type). The combined force–displacement relationship is:

(14) $$\left[\begin{array}{c} \epsilon_{in2}\\ \epsilon_{final2} \end{array}\right]=\left[\begin{array}{cc} C_{12}^{11} & C_{12}^{12}\\ C_{12}^{21} & C_{12}^{22} \end{array}\right]\left[\begin{array}{c} F_{in1}\;\\ F_{out2} \end{array}\right]$$

where $C_{12}^{11}=C_{dia}+[Ad{]}_{12}^{T}C_{bridge}^{11}[Ad{]}_{12}$, $C_{12}^{12}=[Ad{]}_{12}^{T}C_{bridge}^{12}$, $C_{12}^{21}=C_{bridge}^{21}[Ad{]}_{12}$, $C_{12}^{22}=C_{bridge}^{22}$.

The force at the output port *Q* of the second stage is balanced with the input force of the third stage:

(15) $$F_{out2}\;+F_{out3}\;=F_{final}\;$$

From the output equation of the second stage:

(16) $$F_{out2\;}={\left(C_{12}^{22}\;\right)}^{-1}(\epsilon_{final}\;-C_{12}^{21}\;F_{in1}\;)$$

The force of the third stage is given by:

(17) $$F_{out3\;}=K_{para}\;\epsilon_{final}\;$$

where *K_para_* is a 3 × 3 symmetric positive definite matrix, whose element *K_i j_* denotes the generalized force required for unit displacement in *j* direction.

The amplification ratio is calculated under the condition of no load (i.e., no external force or external torque at the output end), and the gripper is only used to transmit displacement without bearing external load.

Under no-load condition (*F*_final_ = 0):

(18) $$\;{\left(C_{12}^{22}\;\right)}^{-1}(\epsilon_{final}\;-C_{12}^{21}\;F_{in1}\;)+K_{para}\;\epsilon_{final}\;=0$$

Rearranging yields:

(19) $$[{\left(C_{12}^{22}\right)}^{-1}+K_{para}]\epsilon_{final}\;={\left(C_{12}^{22}\;\right)}^{-1}C_{12}^{21}\;F_{in1}\;$$

Multiplying both sides by $C_{12}^{22}$ on the left:

(20) $$\;\;[I+C_{12}^{22}\;K_{para}\;]\epsilon_{final}\;=C_{12}^{22}\;\;F_{in1}\;$$

Thus:

(21) $$\;\;\;\epsilon_{final}\;={\left[I+C_{12}^{22}\;K_{para}\;\right]}^{-1}C_{12}^{22}\;\;F_{in1}\;$$

The input displacement of the first stage is:

(22) $$u_{in}\;={\left[C_{dia}\right]}_{11}F_{in1}$$

Therefore, the total DAR of the micromanipulator is:

(23) $$R_{\mathrm{amp}}=\frac{u_{final}}{u_{in}}\;=\frac{{\left[{\left(I+C_{12}^{22}K_{para}\right)}^{-1}C_{12}^{21}\right]}_{11}}{{\left[C_{dia}\right]}_{11}}$$

### 3.2. Dynamic Modeling

In addition to the static displacement amplification characteristics, the dynamic performance of the micromanipulator is essential for high-precision operations. The first natural frequency is the most critical dynamic parameter, as it determines the working bandwidth and the ability to avoid external excitation resonance.

The total kinetic energy of the system is:

(24) $$T=\frac{1}{2}\;\sum_{i=1}^{N}m_{i}\;{\dot{u}}_{i}^{2}\;\;+\frac{1}{2}\;\sum_{j=1}^{M}I_{j}\;{\dot{\theta }}_{j}^{2}\;\;\;$$

where *m_i_* and *I_j_* are the mass and rotational inertia of each element, ${\dot{u}}_{i}$ and ${\dot{\theta }}_{j}$ are the corresponding translational and angular velocities. By normalizing all velocities to the input velocity ${\dot{x}}_{in}$, the equivalent mass is obtained as:

(25) $$\;M_{eq}\;=\sum_{i=1}^{N}m_{i}\;{\left(\frac{{\dot{u}}_{i}}{{\dot{x}}_{in}}\right)}^{2}+\;\sum_{j=1}^{M}I_{j}\;{\left(\frac{{\dot{\theta }}_{j}}{{\dot{x}}_{in}}\right)}^{2}{\dot{\theta }}_{j}^{2}$$

The equivalent stiffness is derived from the strain energy of the flexure hinges. For a rectangular flexure hinge under bending, the bending stiffness is:

(26) $$\;k_{bend}\;=\frac{Ebt^{3}}{12l}\;$$

where *E* is the elastic modulus. For an axially deformed hinge, the axial stiffness is:

(27) $$\;k_{axial}\;=\frac{Ebt}{l}\;$$

Summing the strain energy contributions of all deformable hinges:

(28) $$U=\frac{1}{2}\;\sum_{p=1}^{P}\left(k_{bend,p}\theta_{p}^{2}+k_{axial,p}\;\Delta_{p}^{2}\right)\;$$

Normalizing the deformations to the input displacement *x*_in_, the equivalent stiffness is:

(29) $$K_{eq}=\;\sum_{p=1}^{P}\left[k_{bend,p}\;{\left(\frac{\theta_{p}}{x_{in}}\right)}^{2}+k_{axial,p}\;{\left(\frac{\Delta_{p}}{x_{in}}\right)}^{2}\right]\;\;$$

The natural frequency is:

(30) $$\;f_{n}\;=\frac{1}{2\pi }\;\sqrt{\frac{M_{eq}}{K_{eq}}}\;\;\;\;$$

## 4. Finite Element Analysis (FEA)

### 4.1. Design Optimization

To improve the DA efficiency of the micromanipulator, systematic optimization of the key structural parameters is required. In this paper, the finite element method combined with the response surface methodology is adopted to optimize the critical dimensional parameters of the micromanipulator. During the optimization process, the main material of the micromanipulator is selected as 7075 aluminum alloy, with the following material parameters: elastic modulus *E* = 71 GPa, Poisson’s ratio *ν* = 0.33, yield strength *σ*_s_ = 455 MPa, and density *ρ* = 2810 kg/m^3^. A parametric finite element model is constructed using ANSYS Workbench 18.0, meshed with hexahedral elements, and full degrees-of-freedom constraints are applied at the fixing bolt holes.

(1) Objective function

The maximum output displacement *S*_out_ of the micromanipulator jaw is taken as the optimization objective: maximize *S*_out_ = *S*_max_

(2) Design variables

Based on the sensitivity analysis, the design variables are categorized into three functional modules: the rhombus mechanism (initial angle *α*, arm thickness *b*_1_), the bridge-type mechanism (arm length *b*_2_, hinge thickness *h*), and the parallelogram mechanism (long arm length *l*_1_, short arm length *l*_2_, hinge length *l*_3_, hinge thickness *b*_3_). The value ranges are determined by structural space constraints and machining feasibility.

(3) Constraints

To ensure that the structure operates within the elastic range and possesses a sufficient safety margin, the following constraints are imposed:

Strength constraint: The maximum equivalent stress *σ*_max_ of the structure shall not exceed 1/*n*_a_ of the material yield strength, with a safety factor *n*_a_ = 2.

**Figure 4:**
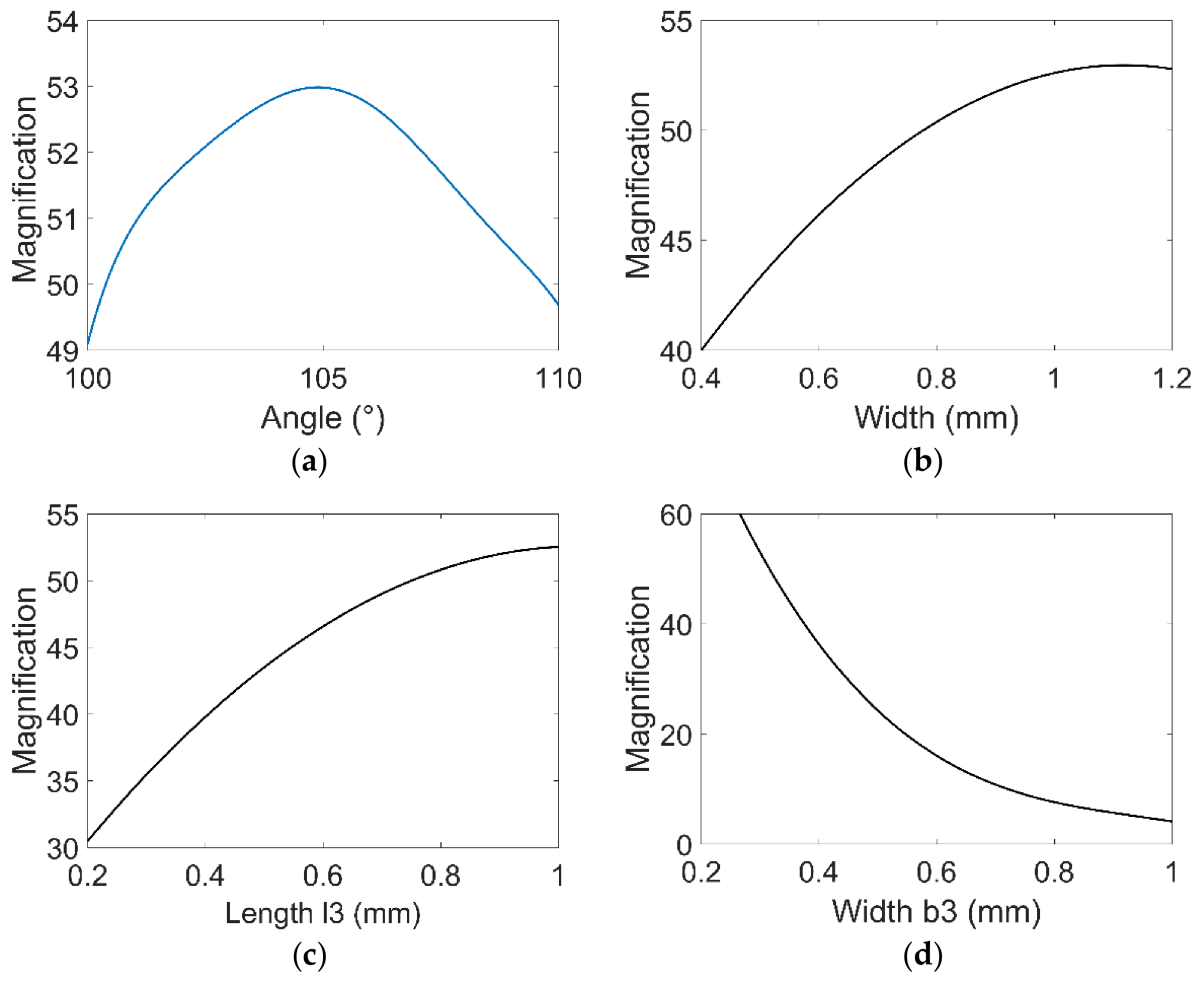

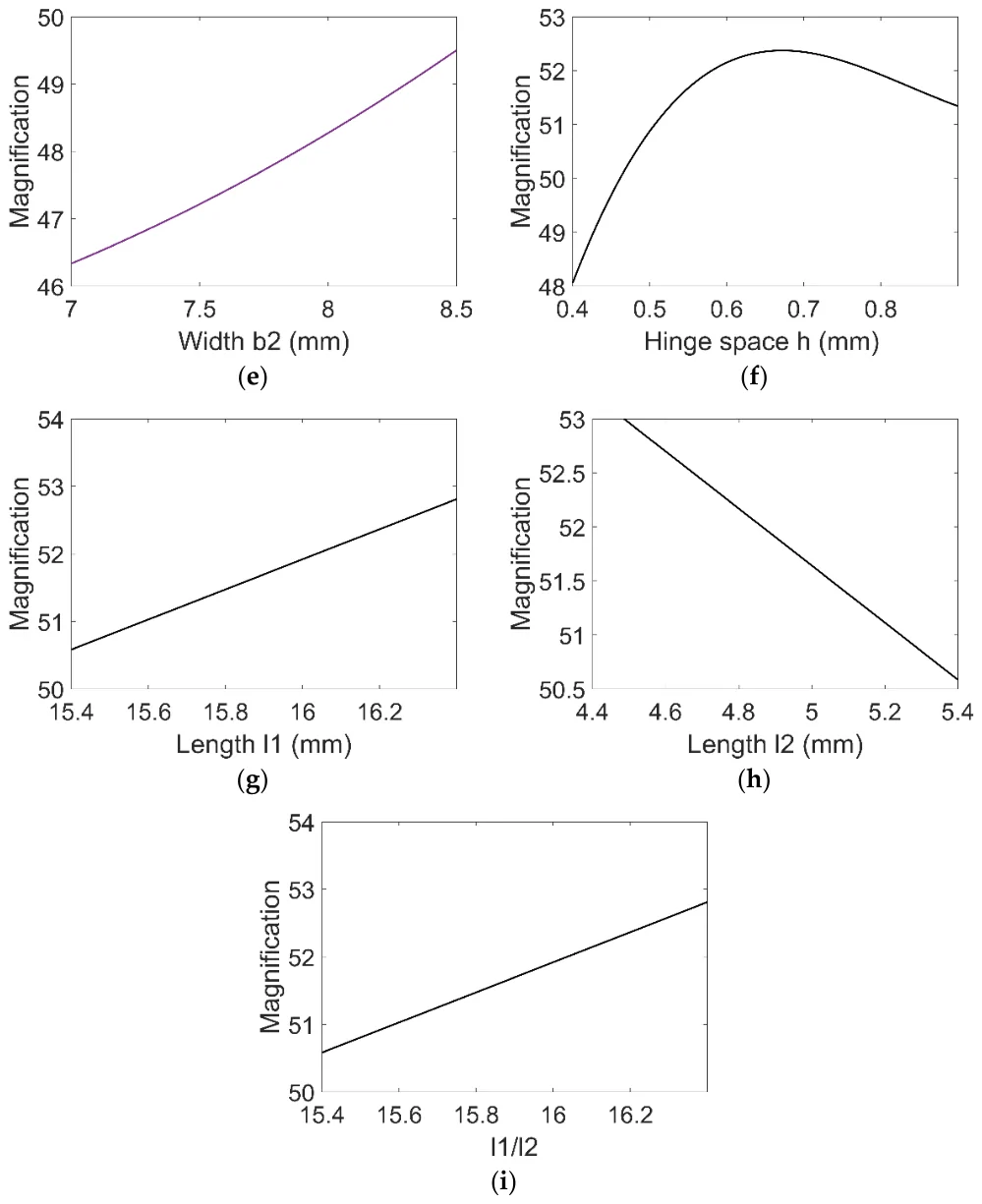
Effects of geometric parameters on the DA factor of the micromanipulator. (**a**) Angle, (**b**) width *b*_1_, (**c**) length *l*_3_, (**d**) width *b*_3_, (**e**) length *b*_2_, (**f**) hinge space *h*, (**g**) length *l*_1_, (**h**) length *l*_2_, and (**i**) *l*_1_/*l*_2_.

Geometric constraints: The allowable ranges of the design variables are summarized in Figure 4, with the specific value ranges defined as follows: 100° ≤ *α* ≤ 110°, 0.4 mm ≤ *b*_1_ ≤ 1.2 mm, 7.0 mm ≤ *b*_2_ ≤ 8.5 mm, 0.4 mm ≤ *h* ≤ 0.89 mm, 15.4 mm ≤ *l*_1_ ≤ 16.4 mm, 4.4 mm ≤ *l*_2_ ≤ 5.4 mm, 0.2 mm ≤ *l*_3_ ≤ 1.0 mm, 0.2 mm ≤ *b*_3_ ≤ 1 mm, 15.4 mm ≤ *l*_1_/*l*_2_ ≤ 16.4 mm.

(4) Optimization strategy and results

To construct the response surface models, a central composite design (CCD) is employed to generate the design points. A total of 80 experimental runs are produced within the design space. For each sample point, the output displacement and the maximum equivalent stress are evaluated through finite element simulations. A trade-off analysis is subsequently conducted to investigate the influence of individual design variables on the objective function. Based on this analysis, the optimal combination of design parameters is identified, which not only satisfies the prescribed strength constraints but also maximizes the output displacement.

### 4.2. Performance Analysis

**Figure 5:**
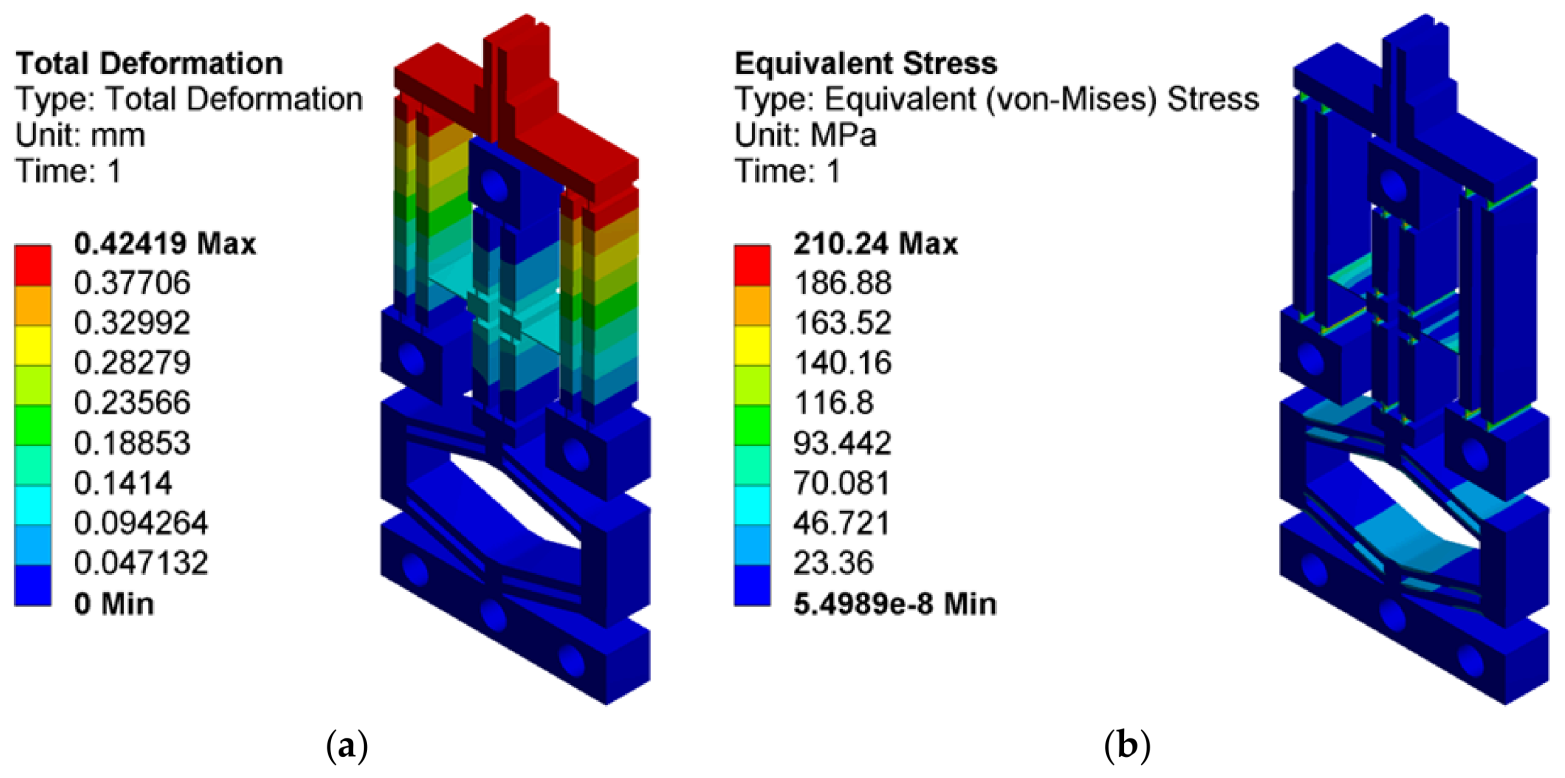
Finite element analysis results of the micromanipulator. (**a**) output displacement, (**b**) equivalent stress.

A finite element analysis is performed on the micromanipulator, in which full degrees-of-freedom fixed constraints are applied to the threaded fixing holes of the micromanipulator to simulate the actual mounting boundary conditions. Figure 5 presents the finite element analysis results of the micromanipulator under static load. An input displacement of 8 μm is applied to the left and right input ends, respectively, to simulate the working state of the piezoelectric actuator. As shown in Figure 5a, the left and right jaws generate symmetrical opening displacements along the x-axis direction, wherein the left jaw moves to the right and the right jaw moves to the left. The calculation results indicate that the total deformation of the left and right jaws is 848.38 μm (i.e., 848.38 μm total opening for both jaws combined). Thus, the DAR of the micromanipulator is 53. Figure 5b shows the equivalent stress distribution of the micromanipulator. The maximum equivalent stress appears in the bending region of the flexure hinges, with a value of 210.24 MPa, which is lower than the material yield strength, indicating that the mechanism possesses sufficient strength margin under normal operating conditions.

In summary, the finite element analysis results validate the excellent performance of the designed micromanipulator in terms of DAR and strength performance, thereby laying a solid foundation for subsequent experimental research.

## 5. Experimental

**Figure 6:**
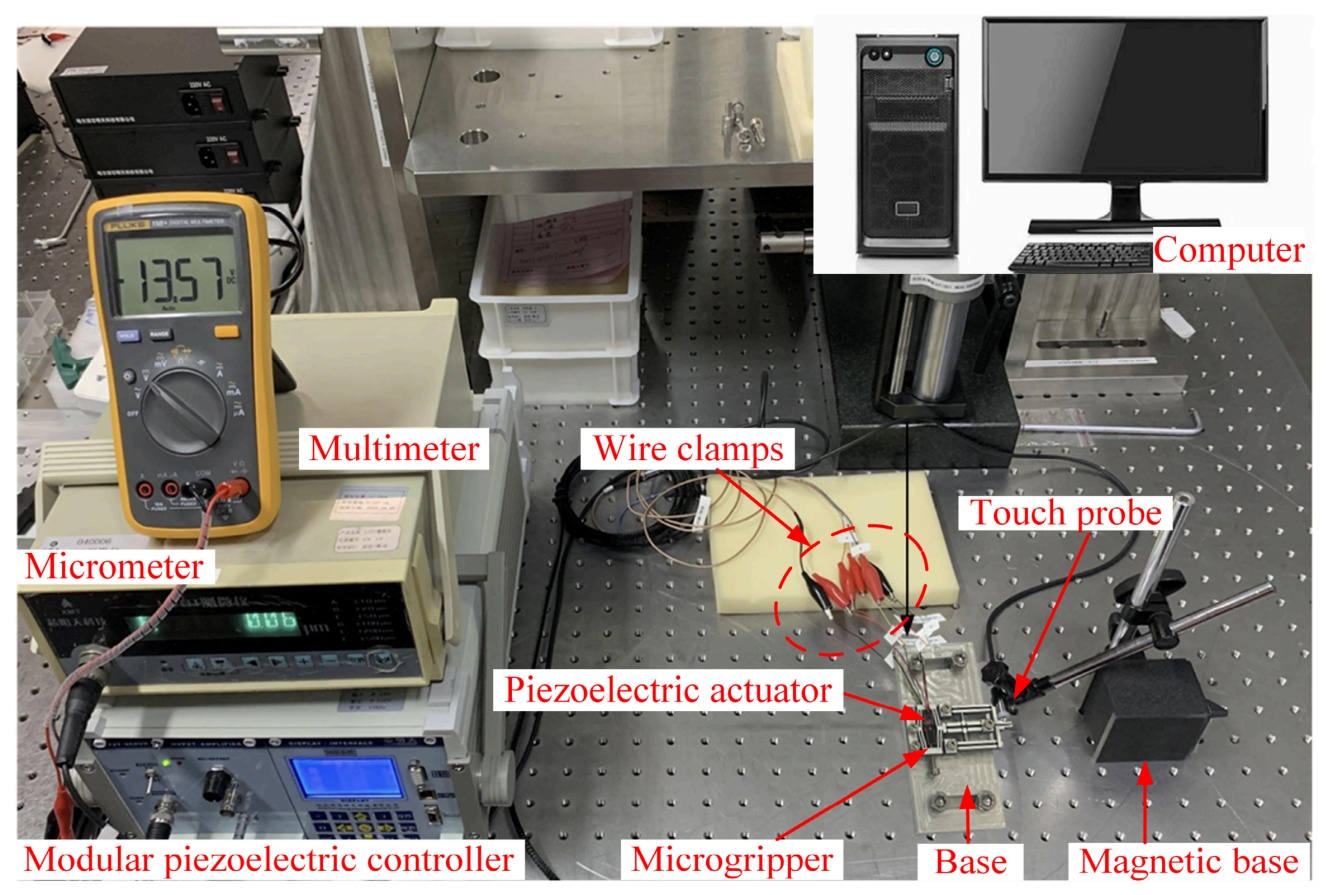
Experimental setup.

To validate the actual performance of the designed micromanipulator, an experimental testing system is established, as shown in Figure 6. This experimental platform mainly comprises the following components: a modular piezoelectric controller, a piezoelectric actuator, resistance strain gauges, a micromanipulator, grasping jaws, a micrometer, a touch probe, a dynamic test instrument, a computer, a base, a magnetic base, wire clamps, as well as a preload bolt and fixing bolts.

Among these components, the piezoelectric actuator serves as the driving unit, which is fixed to the input end of the micromanipulator via the preload bolt, and the driving voltage is provided by the modular piezoelectric controller. Resistance strain gauges are attached to the surface of the piezoelectric actuator to monitor the output displacement of the actuator in real time and provide feedback to the controller for closed-loop control. The micromanipulator is reliably mounted onto the base through the fixing bolts. A magnetic base is placed underneath the touch probe to ensure the stability of the entire device during the experiment. For output displacement measurement, a contact-type LVDT micrometer (Core Tomorrow, Harbin, China,) is employed to perform contact measurement of the tip displacement of the grasping jaws, and the sampling rate is set to 100 Hz during the displacement calibration tests. The dynamic test instrument is used to acquire the dynamic response signals of the micromanipulator and transmit the data to the computer for subsequent processing and analysis. Wire clamps are adopted to organize and secure the connecting cables, thereby avoiding measurement errors induced by cable sway during the experiment. All equipment is installed on a high-performance vibration isolation platform to eliminate the interference of external vibrations on the experimental results. The establishment of this experimental platform provides a reliable foundation for subsequent static and dynamic performance testing.

**Figure 7:**
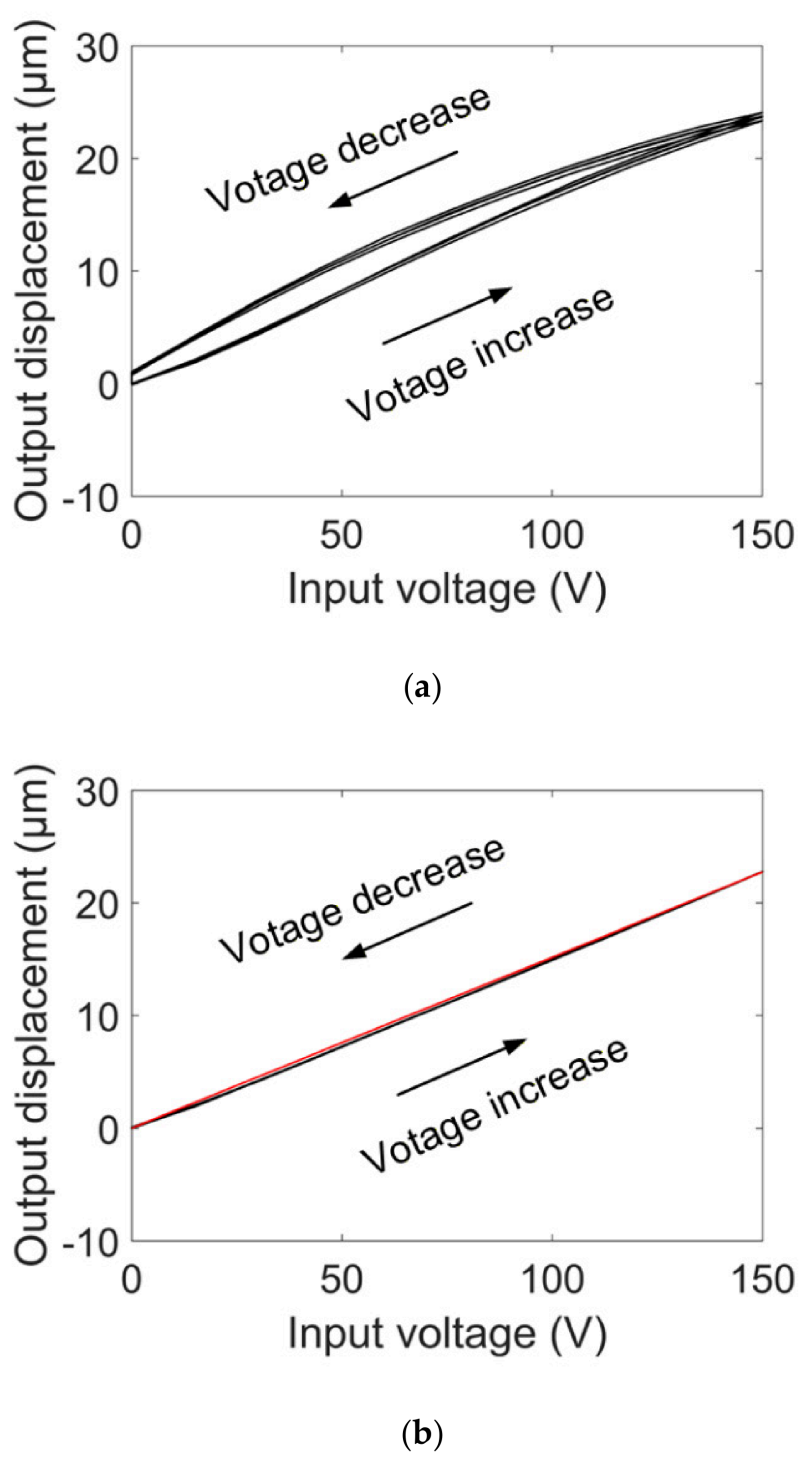

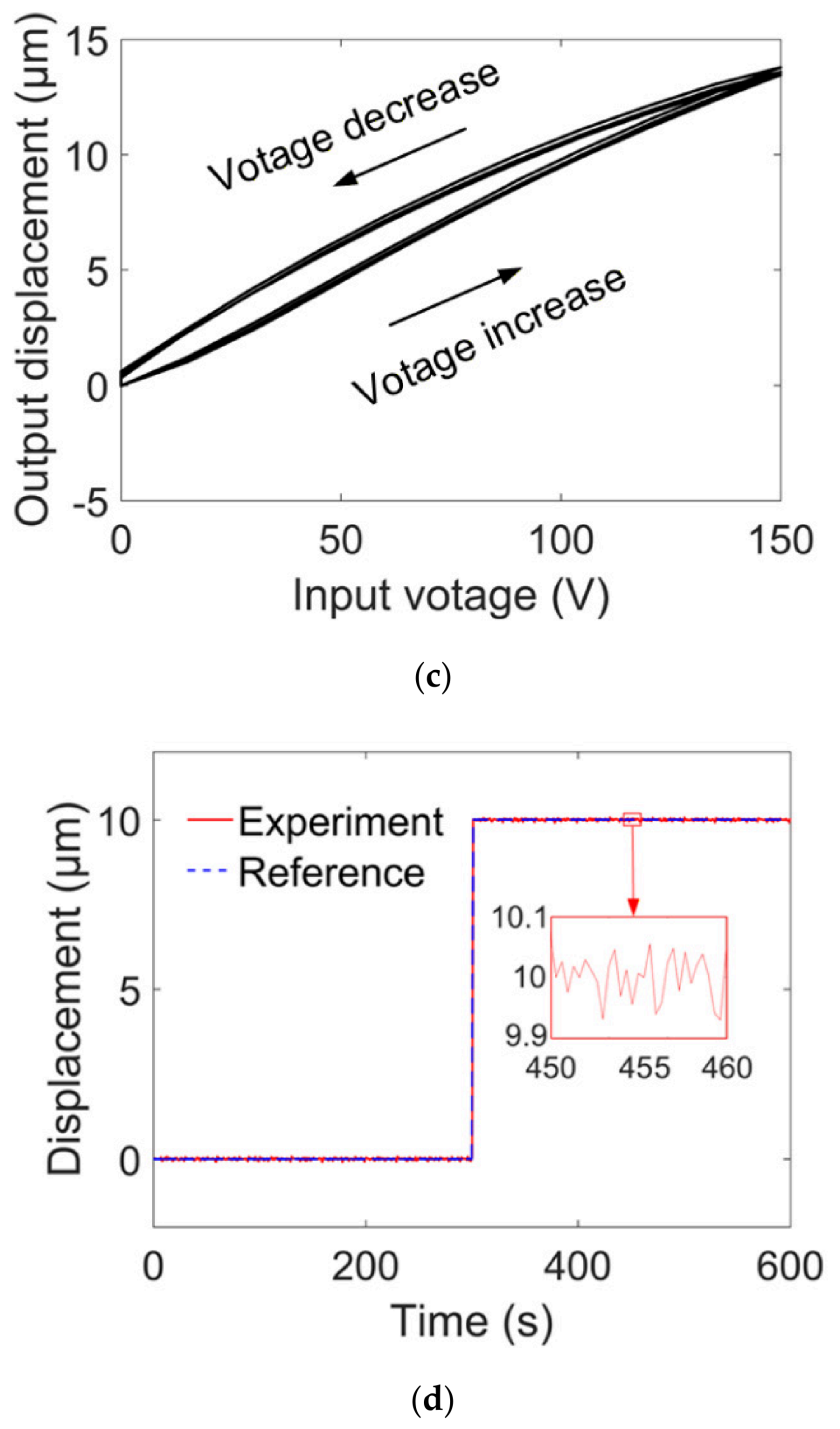
Control characteristics: (**a**) open-loop voltage–displacement curve, (**b**) closed-loop voltage–displacement curve, (**c**) open-loop voltage–displacement curve, and (**d**) closed-loop step response.

Figure 7a presents the relationship curve between the driving voltage and the output displacement of the piezoelectric actuator under open-loop control. In the figure, the horizontal axis represents the driving voltage ranging from 0 to 150 V, and the vertical axis represents the actuator output displacement. The curve consists of two trajectories, corresponding to extension and retraction, respectively. The two trajectories separate from each other, exhibiting pronounced hysteresis characteristics, while the displacement variation also demonstrates nonlinear behavior. This result indicates that under open-loop control, the piezoelectric ceramic actuator exhibits significant hysteresis and creep phenomena, leading to poor output positioning accuracy, which cannot satisfy the high-precision operation requirements of the micromanipulator. Figure 7b shows the relationship curve between the driving voltage and the output displacement under closed-loop control. The coordinate axis parameters are consistent with those in the open-loop control test. After adopting closed-loop control, the two curves corresponding to extension and retraction substantially coincide, the hysteresis effect of the piezoelectric ceramic is effectively suppressed, the linearity of the relationship between voltage and displacement is greatly improved, and the output accuracy and stability of the actuator are significantly enhanced. Figure 7c shows the relationship between the driving voltage of the piezoelectric actuator and the output displacement of the diamond mechanism under open-loop control. The total output displacement of the rhombus mechanism is defined as the sum of the deformation at both ends. By adjusting the output voltage of the piezoelectric controller during the test, the input displacements on the left and right sides of the amplification mechanism are measured separately. The total output displacement of the rhombus mechanism is then obtained by summing the displacement values on both sides. Figure 7d illustrates the tracking response of the closed-loop control system to a 10 μm step displacement command. It can be observed that the actual displacement tracks the reference signal rapidly and accurately, exhibiting no significant steady-state error after reaching the target position. This indicates that the closed-loop control effectively suppresses the creep characteristics of the piezoelectric ceramic and external disturbances, achieving high-precision and high-stability positioning control.

**Figure 8:**
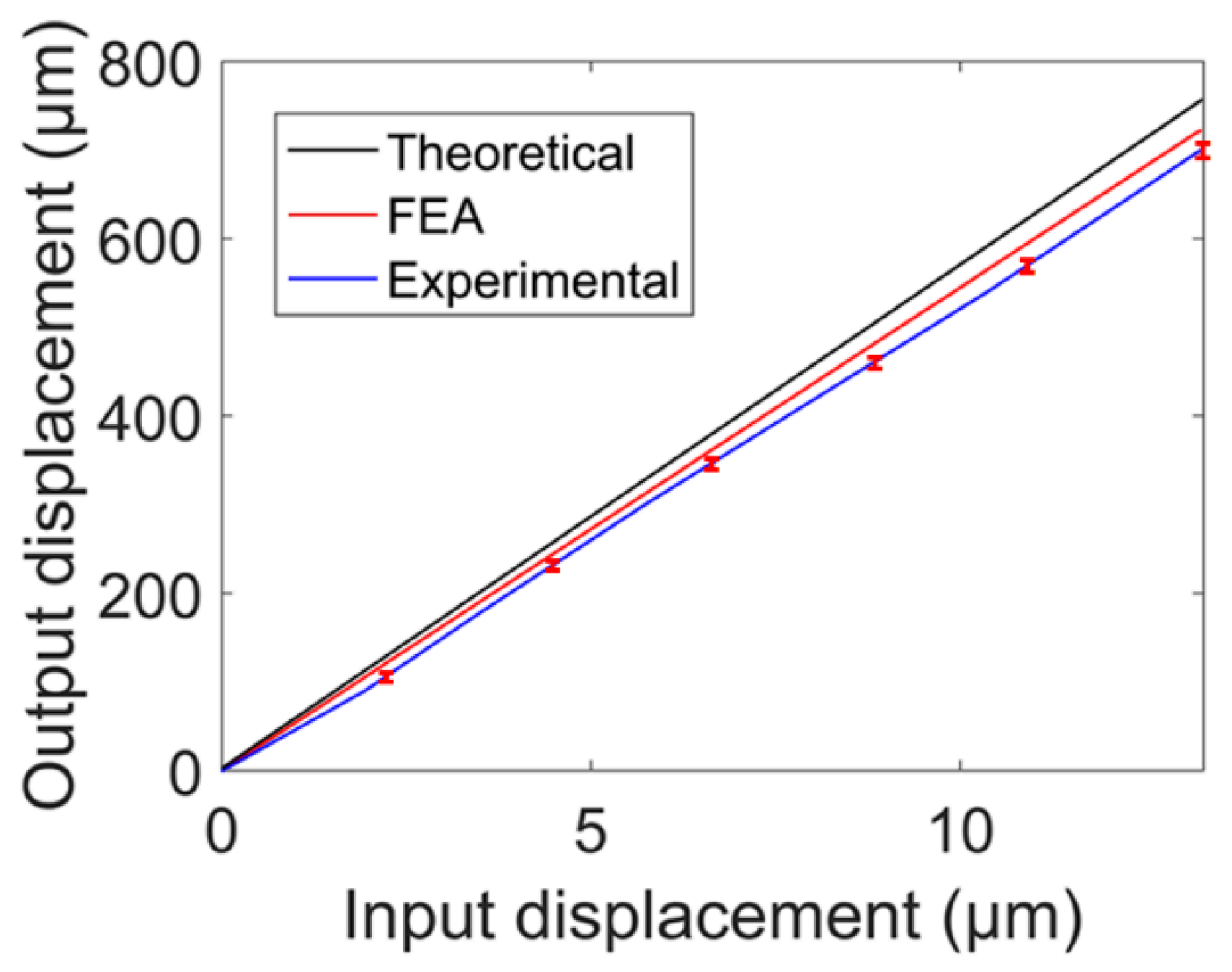
Input-output displacement curve of the micromanipulator.

Figure 8 shows the relationship curve between the input displacement and the output displacement of the micromanipulator. Each measurement was repeated five times, and the mean values with standard deviation error bars are presented in Figure 8. The maximum standard deviation observed across all measurement points is ±5 μm. The curve is approximately linear overall, reflecting that the three-stage compliant amplification mechanism possesses favorable linear transmission characteristics between the input and output displacements. According to the measurement results, the overall DAR predicted by the finite element simulation is 53.0, while the experimentally measured DAR is 51.2, yielding a relative deviation of approximately 3.4%. This level of deviation is within the acceptable range for compliant mechanisms and can be primarily attributed to the following factors: (1) manufacturing tolerances in the dimensions of the flexure hinges, which directly affect the structural compliance and thus the actual amplification ratio; (2) inherent measurement uncertainties in the experimental setup, including sensor resolution, alignment errors, and environmental disturbances. Although the finite element simulation provides a close approximation under idealized assumptions, the experimental results are considered more reliable as they reflect the actual performance of the fabricated prototype under real-world operating conditions. Nevertheless, the strong consistency between simulation and experiment (with a deviation well within 5%) mutually validates both approaches and confirms the effectiveness of the proposed design and optimization methodology. Furthermore, the theoretically derived DAR based on the analytical model is 55.4, which deviates from the experimental result by approximately 8.2%.

To further validate the performance advantages of the micromanipulator proposed in this paper, Table 1 summarizes representative three-stage amplification micromanipulators reported in existing studies and compares their dimensions and amplification ratios. From the data presented in the table, it can be observed that compared with similar mechanisms reported in the literature [20,21,22,23,24], the micromanipulator designed in this paper achieves a significant improvement in the DAR. The DAR of all micromanipulators listed in this table is defined as the total output displacement of the clamping jaws divided by the total elongation of the piezoelectric actuator, which is consistent with the definition adopted in this work.

## 6. Conclusions

In this paper, a three-stage DA micromanipulator is designed, which is composed of a compound rhombus mechanism, a bridge-type mechanism, and a parallelogram mechanism connected in series. By establishing an analytical model of the DAR that accounts for inter-stage stiffness matching and force transmission relationships, the overall displacement transmission law of the series combination of the stages is revealed. On this basis, with the objective of maximizing the output displacement, geometric parameters such as hinge thickness, hinge length, and cross-sectional dimensions of key links are selected as design variables. Considering material strength and structural space constraints, surrogate models are constructed using response surface methodology and central composite design, and a systematic structural size optimization is carried out. The three-stage series amplification configuration, through rational stiffness matching and size optimization, achieves a stepwise increase in the displacement ratio while maintaining a compact structure and translational gripping output. Experimental results show that the maximum output displacement of the micromanipulator is 681 μm, and the DAR of the mechanism reaches 51.2. Compared with existing similar three-stage amplification micromanipulators, the present design achieves a remarkable ratio enhancement under a compact structure. This research validates the effectiveness of the analytical modeling and response surface optimization method.

## Figures and Tables

**Table 1:** Performance comparison of micromanipulators.

Micromanipulator	Size (mm^2^)	Magnification	Stage
Reference [19]	31.3 × 51.7	5.2	Single-stage
Reference [20]	22 × 62	11.19	Three-stage
Reference [21]	-	20.1	Three-stage
Reference [22]	>25 × 50	12.76	Three-stage
Reference [23]	>22 × 64	33.6	Three-stage
Reference [24]	>38 × 58	22.8	Three-stage
This work	26 × 58.2	53	Three-stage

## Data Availability

The data presented in this study are available on request from the authors.
